# Supplementary figures and images for: LINflow: a computational pipeline that combines an alignment-free with an alignment-based method to accelerate generation of similarity matrices for prokaryotic genomes
Source: PeerJ. 2021 Mar 24;9:e10906. doi: 10.7717/peerj.10906 (PMC8000461; doi:10.7717/peerj.10906)

**Supplementary Figure 1.** Heatmap based on the ANI matrix calculated by pyani for data set A .

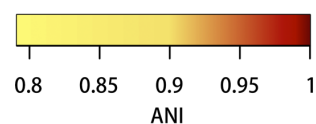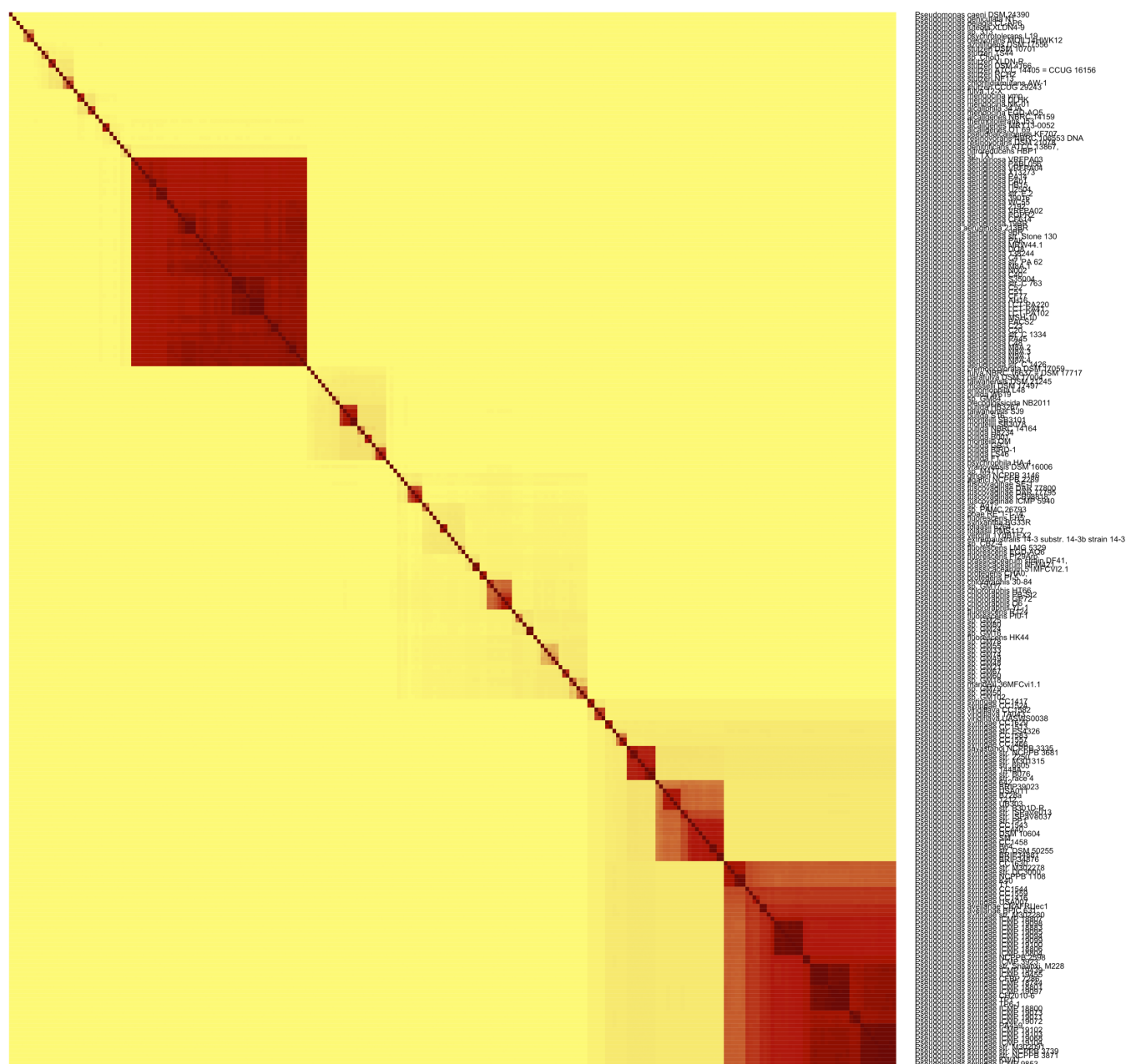

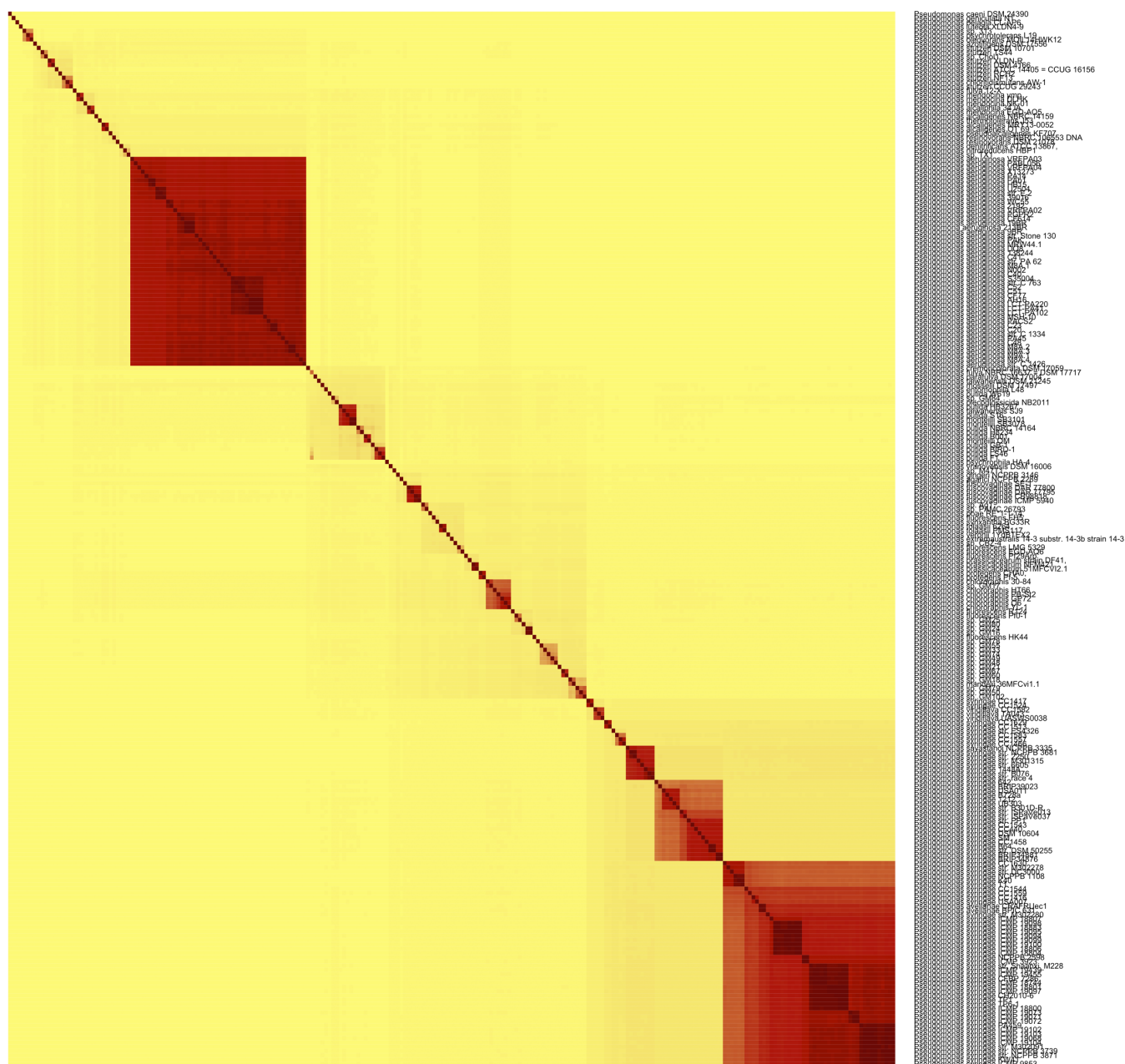

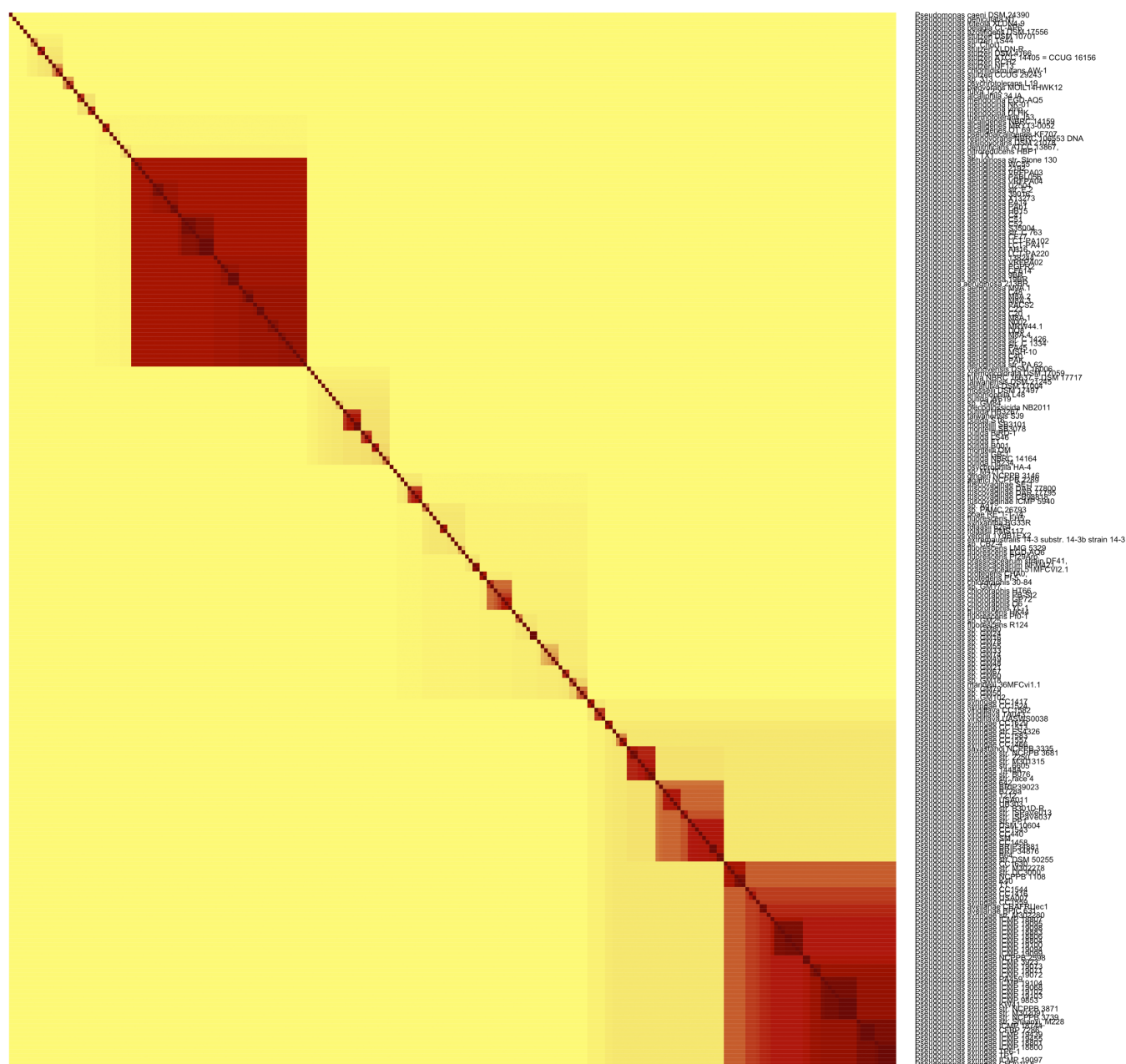

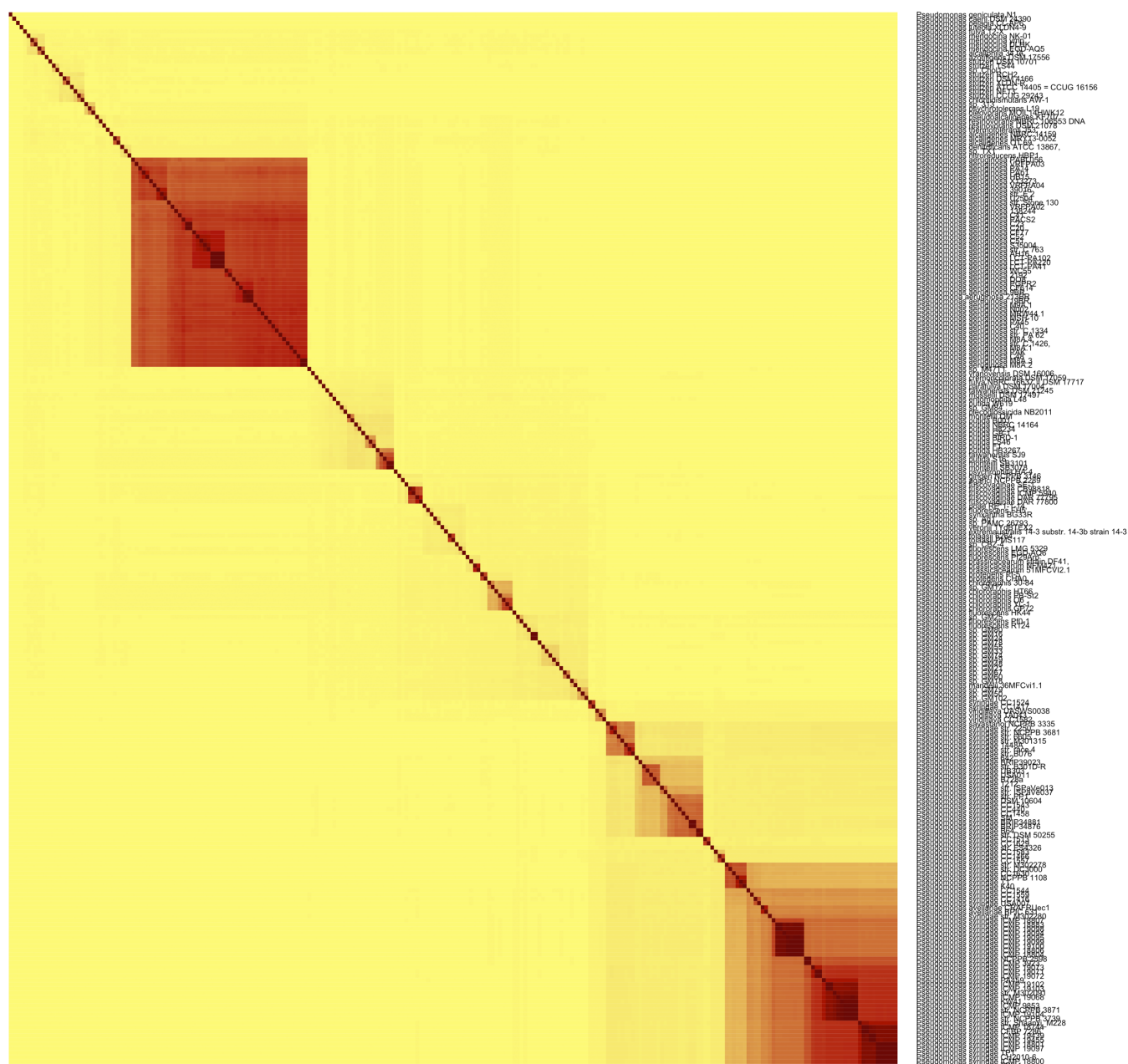

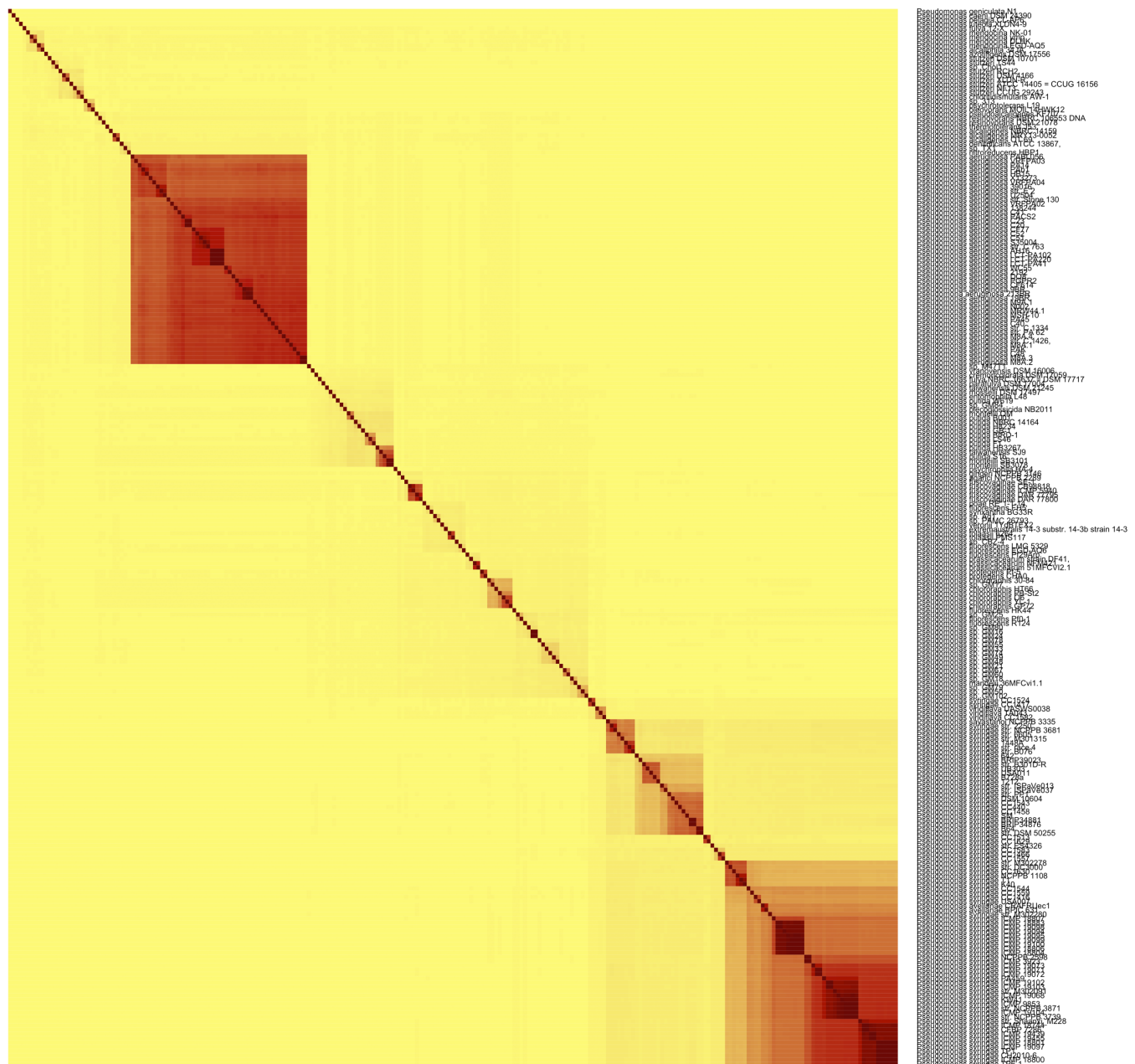

Supplement: Supplemental Information 1 — Figures 1 through 5 correspond to panels A through E in Fig. 3 but include strain names. [file peerj-09-10906-s001.pdf]
