# Supplementary material for: LINflow: a computational pipeline that combines an alignment-free with an alignment-based method to accelerate generation of similarity matrices for prokaryotic genomes
Source: PeerJ. 2021 Mar 24;9:e10906. doi: 10.7717/peerj.10906 (PMC8000461; doi:10.7717/peerj.10906)

Supplementary Figure 16. Heatmap based on the ANI matrix calculated by pyani for data set D .

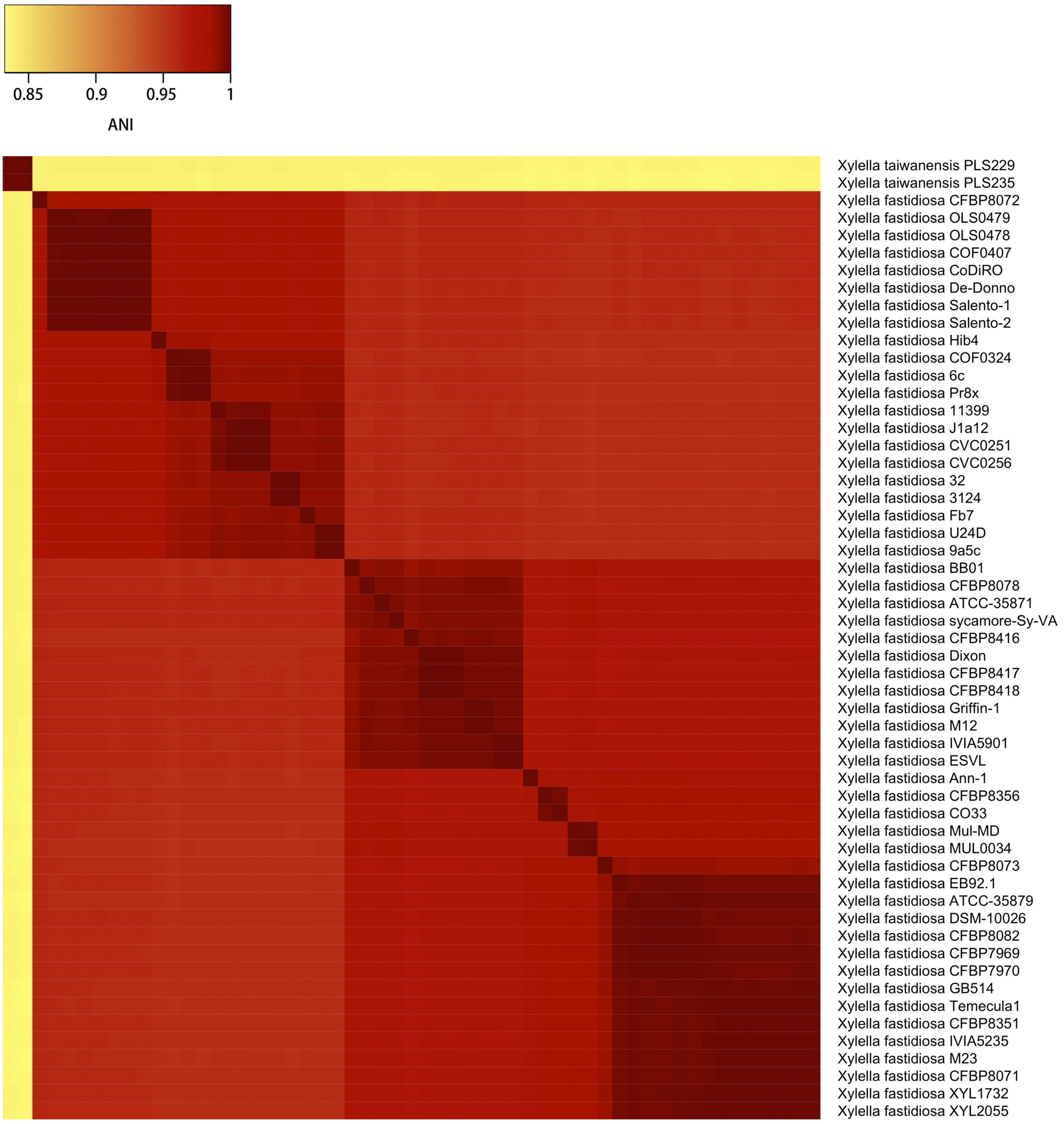

Supplementary Figure 17. Heatmap based on the ANI matrix calculated by FastANI for data set D.

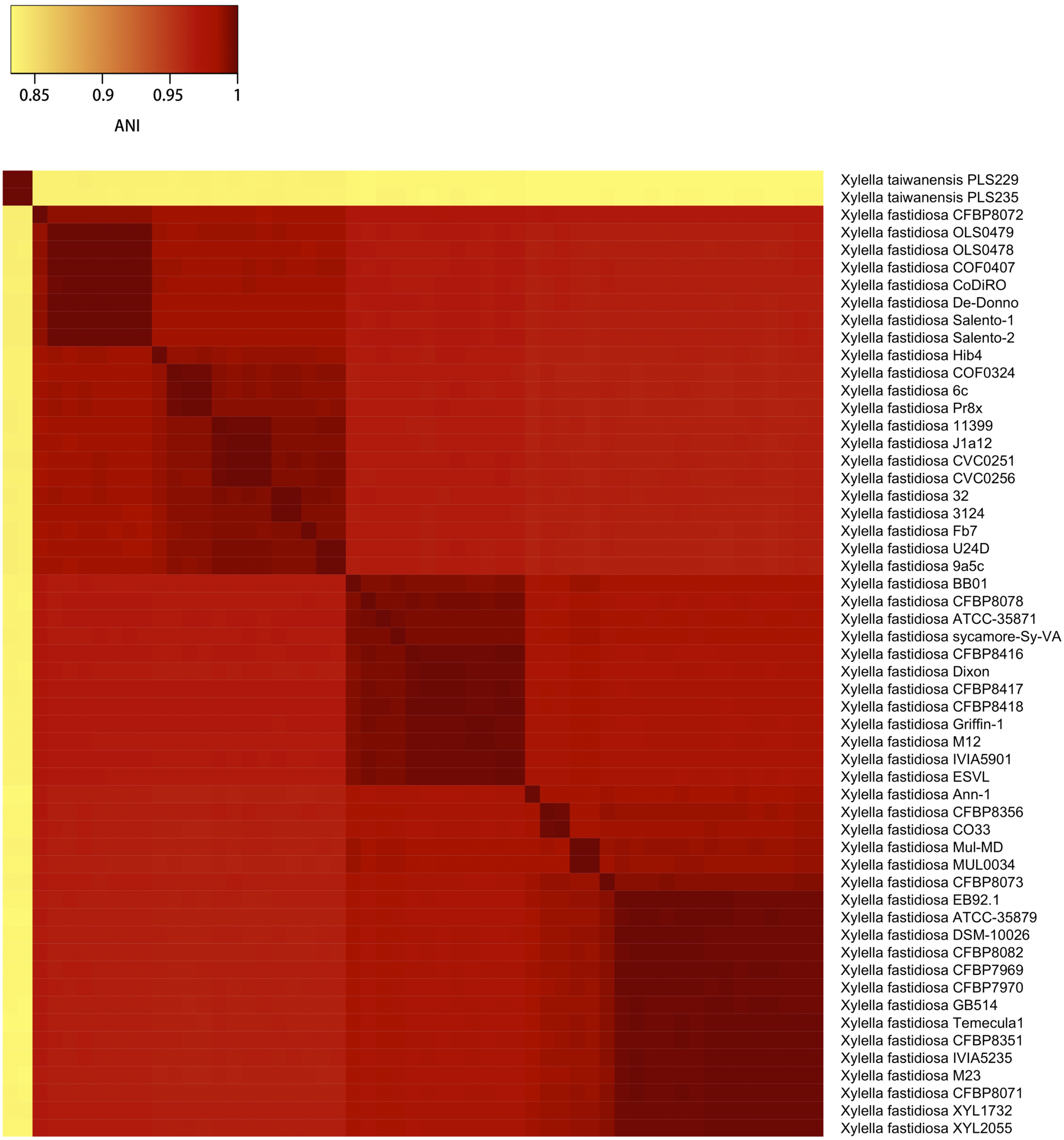

Supplementary Figure 18. Heatmap based on the ANI matrix calculated by LINflow for data set D.

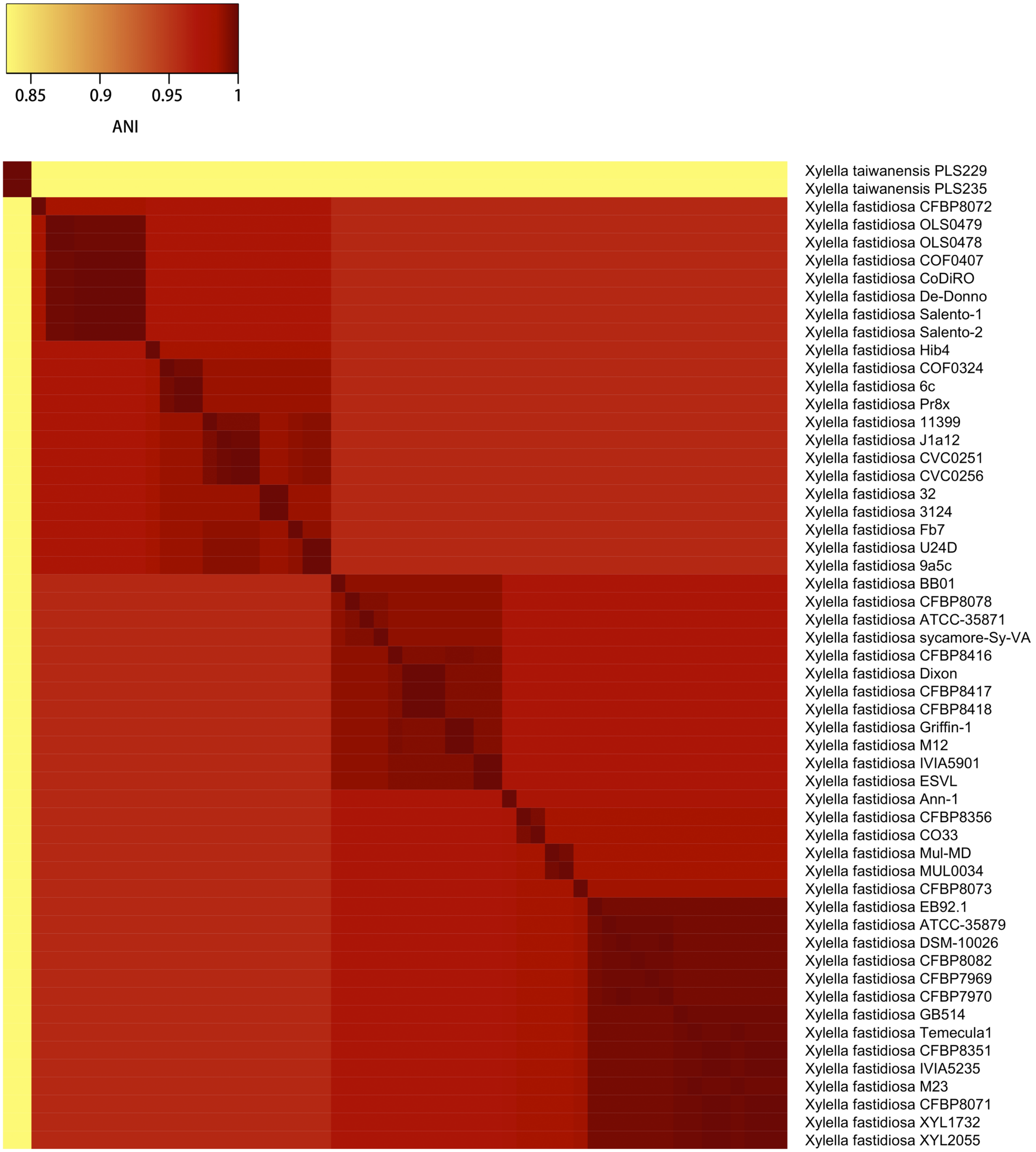

Supplementary Figure 19. Heatmap based on the Jaccard similarity matrix calculated by sourmash with k=21 for data set D.

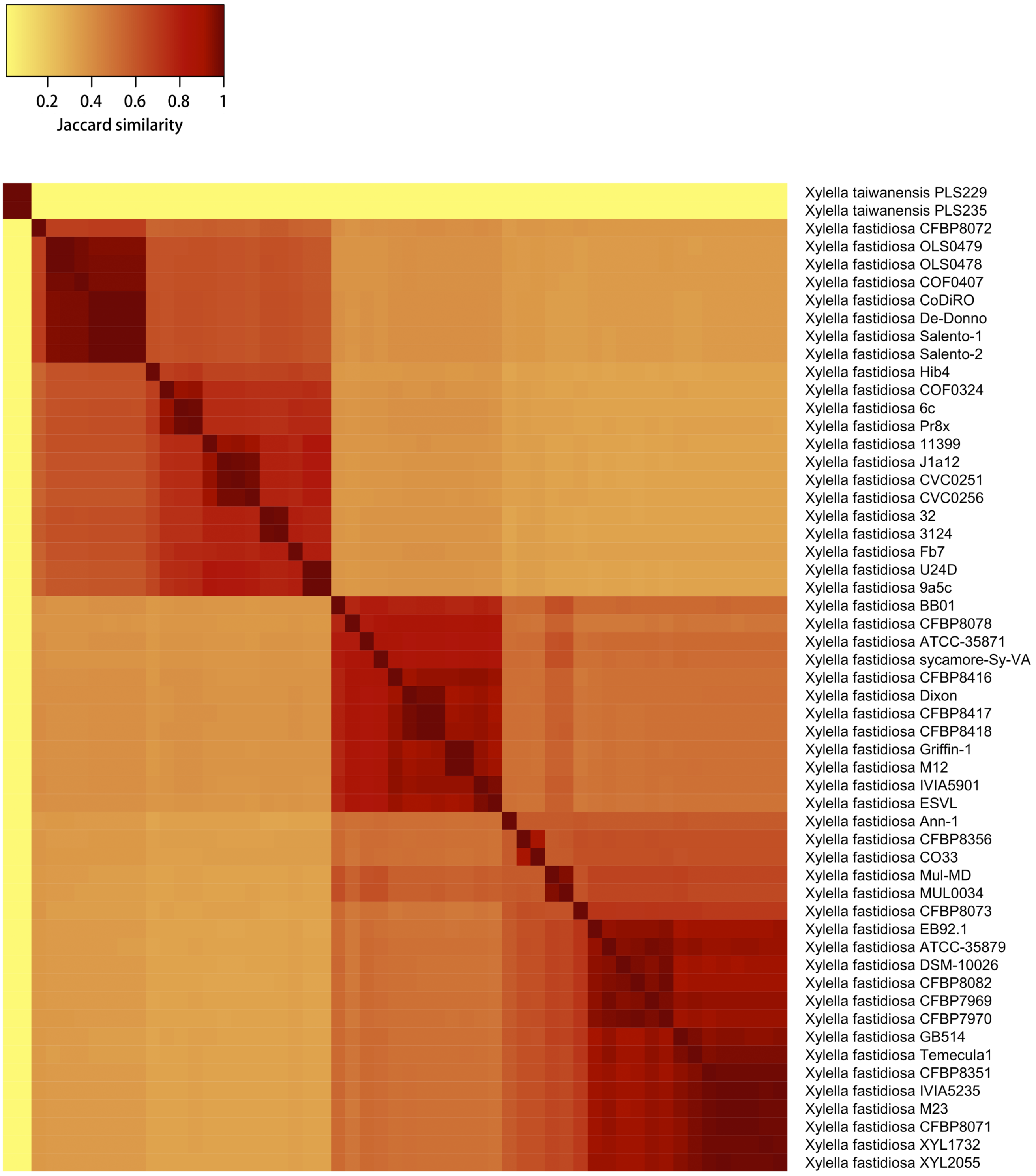

Supplementary Figure 20. Heatmap based on the Jaccard similarity matrix calculated by sourmash with k=51 for data set D.

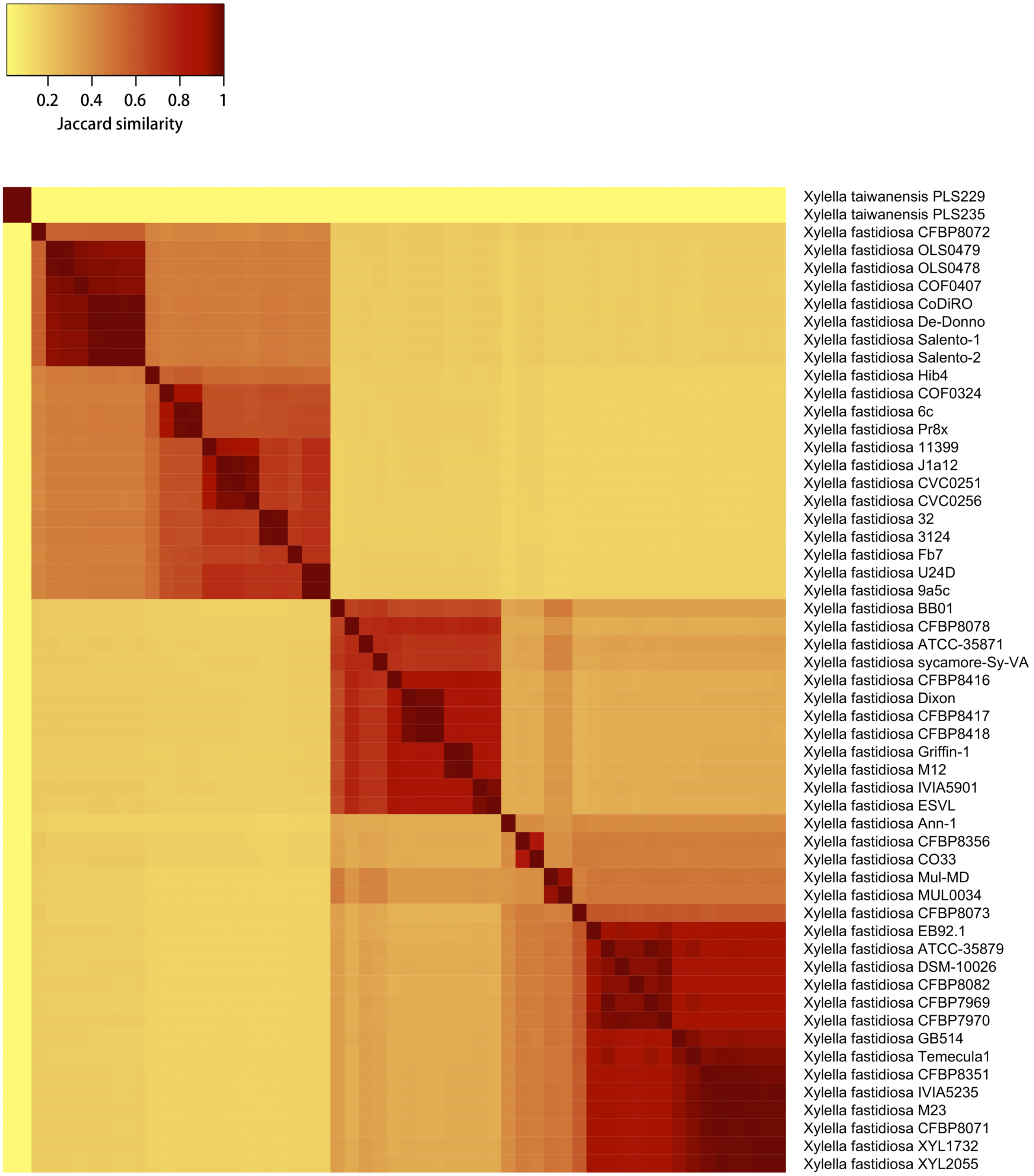

Supplement: Supplemental Information 4 — Heatmaps based on hierarchical clustering using the complete linkage method using the similarity matrices obtained with pyani (SF. 16), FastANI (SF. 17), LINflow (SF. 18), Sourmash k = 21 (SF. 19) and k = 51 (SF. 20) for dataset D. [file peerj-09-10906-s004.pdf]
